# Interrogating differences in expression of targeted gene sets to predict breast cancer outcome

**DOI:** 10.1186/1471-2407-13-326

**Published:** 2013-07-02

**Authors:** Sarah A Andres, Guy N Brock, James L Wittliff

**Affiliations:** 1 Hormone Receptor Laboratory, Department of Biochemistry & Molecular Biology, Brown Cancer Center and the Institute for Molecular Diversity & Drug Design, University of Louisville, Louisville, KY 40292, USA; 2 Department of Bioinformatics and Biostatistics, University of Louisville, Louisville, KY 40292, USA

**Keywords:** Breast cancer, Invasive ductal carcinoma, Risk of recurrence, Prognostic test

## Abstract

**Background:**

Genomics provides opportunities to develop precise tests for diagnostics, therapy selection and monitoring. From analyses of our studies and those of published results, 32 candidate genes were identified, whose expression appears related to clinical outcome of breast cancer. Expression of these genes was validated by qPCR and correlated with clinical follow-up to identify a gene subset for development of a prognostic test.

**Methods:**

RNA was isolated from 225 frozen invasive ductal carcinomas,and qRT-PCR was performed. Univariate hazard ratios and 95% confidence intervals for breast cancer mortality and recurrence were calculated for each of the 32 candidate genes. A multivariable gene expression model for predicting each outcome was determined using the LASSO, with 1000 splits of the data into training and testing sets to determine predictive accuracy based on the C-index. Models with gene expression data were compared to models with standard clinical covariates and models with both gene expression and clinical covariates.

**Results:**

Univariate analyses revealed over-expression of RABEP1, PGR, NAT1, PTP4A2, SLC39A6, ESR1, EVL, TBC1D9, FUT8, and SCUBE2 were all associated with reduced time to disease-related mortality (HR between 0.8 and 0.91, adjusted *p *< 0.05), while RABEP1, PGR, SLC39A6, and FUT8 were also associated with reduced recurrence times. Multivariable analyses using the LASSO revealed PGR, ESR1, NAT1, GABRP, TBC1D9, SLC39A6, and LRBA to be the most important predictors for both disease mortality and recurrence. Median C-indexes on test data sets for the gene expression, clinical, and combined models were 0.65, 0.63, and 0.65 for disease mortality and 0.64, 0.63, and 0.66 for disease recurrence, respectively.

**Conclusions:**

Molecular signatures consisting of five genes (PGR, GABRP, TBC1D9, SLC39A6 and LRBA) for disease mortality and of six genes (PGR, ESR1, GABRP, TBC1D9, SLC39A6 and LRBA) for disease recurrence were identified. These signatures were as effective as standard clinical parameters in predicting recurrence/mortality, and when combined, offered some improvement relative to clinical information alone for disease recurrence (median difference in C-values of 0.03, 95% CI of -0.08 to 0.13). Collectively, results suggest that these genes form the basis for a clinical laboratory test to predict clinical outcome of breast cancer.

## Background

Our goal is to associate patient-related characteristics and treatment outcome, tumor pathology and biomarker status with newly derived information from genomic and proteomic studies to advance the theranostics of breast carcinoma. Cellular heterogeneity of tissue specimens has been a complicating factor in determining analyte (protein or gene) levels in specific cell types, e.g., [1-4]. Numerous studies, including our own, have reported a “molecular signature” of different cancer types, including breast cancer. However, there is great variation in methods utilized to obtain these gene expression profiles, including the use of breast cancer cell lines [5], whole tissue extraction [6-11] and laser capture microdissection (LCM) procured cells [12-15]. In order to obtain a clinically relevant gene set for breast cancer, our hypothesis was examined under the premise that a particular gene should be present in multiple gene expression profiles despite the differences in methodology used to determine the molecular signature. By data-mining these studies collectively, a gene set was compiled and analyzed for clinical utility in breast cancer patients.

In this study, we constructed Cox proportional hazards [16-18] models to predict risk of disease recurrence and overall survival, using a selected panel of candidate biomarkers with suspected association with breast cancer outcomes. To rigorously develop our models, we used the least absolute shrinkage and selection operator (LASSO) [19] for variable selection and evaluated their predictive ability using repeated splits of the data into training and test sets. Models based on gene expression are compared with models based on clinical information to evaluate the gain in predictive accuracy over standard clinical management parameters. Our ultimate goal is to develop a clinically-relevant gene expression-based test for use in hospital laboratories, in order to assist in clinical decisions improving breast cancer management, as well as gain insight into the interrelationships between the genes and clinical outcome of breast cancer patients. Our approach includes the identification of new molecular targets for drug design and developing companion diagnostics.

## Methods

The investigations described were part of a study that is approved by the Human Subject Protection Program Institutional Review Board at the University of Louisville. A unique IRB-approved Database and Biorepository composed of de-identified tissue specimens previously collected under stringent conditions [20] for clinical assays of estrogen (ER) and progestin receptors (PR) were used. De-identified specimens of primary invasive ductal carcinoma of the breast obtained from tissue biopsies collected from 1988–1996 were examined using REMARK criteria [21]. Germaine to our studies (e.g., [22,23]), analyses of ER and PR were performed by FDA-approved methods quantifying levels of these clinical biomarkers under stringent quality control measures (e.g., [20,24]) unlike the majority of reports that used immuno-histochemical analyses prior to the release of the College of American Pathologists/American Society of Clinical Oncology (CAP/ASCO) Guidelines [25]. Patients were treated with the standard of care at the time of diagnosis. Tissue-based properties (e.g., pathology, grade, size, and tumor marker expression) and patient-related characteristics (e.g., age, race, smoking status, menopausal status, stage, and nodal status) were utilized to determine relationships between gene expression and clinical parameters. A retrospective analysis of frozen tissue specimens from 225 biopsies of invasive ductal carcinoma was performed (Additional file 1: Figure S1). De-identified clinical and pathological characteristics for each patient evaluated in the study are included in Additional file 2: Table S1. Tissue sections utilized for analyses of gene expression contained a median of 60% breast carcinoma cells (range of 10-95%) and 25% stromal cells (range of 5-65%).

### Gene list selection

In order to obtain a clinically relevant gene set for this investigation, our hypothesis was that a particular gene should be present in multiple gene expression profiles of breast cancer despite the differences in methodology used to determine the molecular signature. GenBank Accession numbers (NCBI) of genes deciphered from our studies using LCM-procured carcinoma cells and those of other published studies [5-14] were entered into the UniGene database (National Center for Biotechnology Information (NCBI)), which separates GenBank sequences into a non-redundant set of gene-oriented clusters. UniGene identifiers for all studies were compiled into Microsoft® Access and analyzed collectively. This comparison identified genes appearing in at least three signatures, generating candidates (EVL, NAT1, ESR1, GABRP, ST8SIA1, TBC1D9, TRIM29, SCUBE2, IL6ST, RABEP1, SLC39A6, TPBG, TCEAL1, DSC2, FUT8, CENPA, MELK, PFKP, PLK1, XBP1, MCM6, BUB1, PTP4A2, YBX1, LRBA, GATA3, CX3CL1, MAPRE2, GMPS and CKS2) for investigating associations with clinical behavior of breast cancer. PGR was also included in the candidate gene list due to its known implications in breast carcinoma [20].

### Gene expression analyses

Levels of mRNA expression were analyzed after isolation with Qiagen (Valencia, CA) RNeasy® RNA isolation kits. Quality of RNA was evaluated with Agilent RNA 6000 Nano Kits and the Bioanalyzer™ Instrument (Agilent Technologies, Palo Alto, CA). Total RNA extracted from the intact tissue section was reverse transcribed in a solution of 250 mMTris-HCl buffer, pH 8.3 containing 375 mMKCl, and 15 mM MgCl_2_ (Invitrogen, Carlsbad, CA), 0.1 M DTT (dithiothreitol, Invitrogen), 10 mMdNTPs (Invitrogen), 20 U/reaction of RNasin™ ribonuclease inhibitor (Promega, Madison, WI) and 200 U/reaction of Superscript™ III RT (reverse transcriptase, Invitrogen) with 5 ng T7 primers. cDNA obtained from this reverse transcription reaction was diluted 10-fold in 2 ng/μl polyinosinic acid and used in qPCR reactions.

qPCR reactions were performed in a 384-well plate using a total volume of 10 μl/well. Reactions contained Power Sybr™ Green PCR Master Mix (Applied Biosystems, Foster City, CA), forward/reverse primers and diluted cDNA obtained from the reverse transcription reaction. Primers were designed with Primer Express™ (Applied Biosystems) to generate sequences closer to the 3’ end of the transcript for use with the oligo (dT) primer in reverse transcription reactions. qPCR reactions were performed in triplicate with duplicate wells in each 384-well plate. Relative gene expression levels were determined using the ΔΔCt method using ACTB for normalization and Universal Human Reference RNA (Stratagene, La Jolla, CA) as the calibrator.

### Power

The power available in this study to detect a hazard ratio of a given magnitude was determined by the following formula, $\log\left(\mathrm{HR}\right)=\sqrt{{\left(z_{1-\alpha }+z_{1-\beta }\right)}^{2}/\left(D\sigma^{2}\right)}$[26]. Here α = 0.05/32, β = 0.2, *z* are quantiles from the standard normal distribution, *D* = 68 is the number of breast-cancer related mortality outcomes, and σ = 1.8 is the median standard deviation of the log_2_ expression values among all 32 genes. The result is that there is 80% power in the current study to detect hazard ratios of 1.116 or larger (equivalently, 0.90 or smaller) per unit increase in log_2_ expression.

### Descriptive statistics and univariate survival analysis

Summary statistics were reported for both gene expression values and clinical covariates. Univariate Cox regression models [16] were fitted to evaluate the association of both gene expression values and clinical covariates with overall and disease-free survival. Calculations and model development were performed using log_2_ transformations of relative gene expression data as determined by qPCR (Additional file 3: Table S2). To account for multiple comparisons, *p*-values were adjusted to control the false-discovery rate (FDR). Because the gene expression values were highly correlated, the method of Benjamini and Yekutieli (BY) [27], which controls for multiple dependent hypothesis tests, was used in lieu of the standard Benjamini and Hochberg (BH) method [28] (the BH method, however, was used for clinical covariates).

### Multivariable Cox models, variable selection, and predictive accuracy

A multivariable Cox proportional hazards model was used to develop a predictive model of overall and disease-free survival, based on the gene expression values and clinical covariates. The model has the following form

$$\lambda \left(t|x_{i}\right)=\lambda_{0}\left(t\right)\exp\left(\sum_{j=1}^{p}x_{\mathrm{ij}}\beta_{j}\right)$$

Where *x*_*1*_*,…,x*_*p*_ are covariates (here, either gene expression values or clinical covariates), *λ(t|x*_*i*_*)* is the hazard at time *t* for the *i*^*th*^ observation, *λ*_*0*_*(t)* is the unspecified baseline hazard function, and $\vec{\beta }=\langle \beta_{1},\ldots ,\beta_{p}\rangle$ is the vector of regression coefficients [29].

Due to the noted shortcomings of stepwise selection strategies [30] and the high correlation between gene expression values, initial variable selection to determine which genes were significant predictors of breast cancer survival and recurrence was done by incorporating a LASSO (least absolute shrinkage and selection operator), or L_1_, penalty [19] on the regression coefficients *β*_1_, … *β*_*p*_. The LASSO penalizes the size of the parameter vector, $\vec{\beta }$ so that unimportant variables (variables whose *β* coefficients are close to zero) are removed from the model. This results in a penalized log partial likelihood function of the form $l\left(\beta \right)-\sum_{j=1}^{p}\lambda \left|\beta_{j}\right|$, where *l*(*β*) denotes the standard Cox log partial likelihood. The maximum likelihood estimates $\hat{\beta }$ are those which maximize this penalized likelihood. The parameter *λ* is the shrinkage parameter and determines the extent of variable selection, with larger values corresponding to a larger penalty and a greater number of variables removed. The optimal value for *λ* was determined using 10-fold cross-validation.

To better assess predictive ability and model performance, we performed 1000 independent splits of the data into training (70%) and test (30%) samples. Splits into training and test samples were stratified on the basis of tumor stage, so that training and test samples were balanced on percent composition of each tumor stage. For each split, a Cox regression model with a LASSO penalty was used to simultaneously fit the model and perform variable selection amongst the 32 genes. For each model, the selected genes and their associated *β* coefficients were recorded, and the number of times that each gene was kept in a model was tabulated. A permutation test was used to calculate a null distribution and determine the significance threshold for the number of times (out of 1000 total permutations) that each gene was retained in a model. Genes with counts above the highest count among the permuted data sets were declared to be significant (roughly corresponding to an empirical p-value of 1/32 = 0.03). Performance of each model was evaluated by the C-index for right-censored data [31], calculated on the test data. The C-index estimates the probability that, for a randomly selected pair of individuals, the individual with the higher risk score (shorter predicted survival time) has the shorter actual event time. Additionally, predictions based on the L_1_-penalized Cox model were used to separate patients in the test data into low and high risk classes based on the linear predictor $\sum_{j=1}^{p}x_{j}\beta_{j}$, with the cut-point for low/high risk based on the median of the linear predictors from the training data. Kaplan-Meier plots based on the original (non-permuted) data were compared to those obtained from the permuted data in order to validate the prognostic significance of the models evaluated.

The selected genes were again used to fit multivariable Cox models based on 1000 independent splits of the data into training and test samples, without any variable selection. C-indexes were calculated for test data predictions based on models fitted to the training data. To assess whether the gene expression values offered any gain in prediction over clinical parameters, models with clinical covariates significantly associated with disease mortality and recurrence were compared with models including both gene expression values and clinical covariates. C-indexes were also calculated separately for ER+ and ER- subsets of breast cancer patients, to assess whether the gene signature was equally effective in each subset. Empirical 95% confidence intervals for the differences in C-indexes between the two sets of models were calculated using the 2.5^th^ and 97.5^th^ percentiles of the differences.

All analyses were performed using R version 2.14.1 [32]. Univariate Cox models were fitted using the R package *survival*[33]*,* while multivariable Cox models with the LASSO penalty were fitted using the *penalized* package [34]. The C-index was calculated using the *rcorrcens* function in the *rms* package [35], and adjustment for multiple comparisons was done using the *multtest* package [36].

### Validation using the TRANSBIG data

Gene expression models for both overall disease survival and recurrence were validated using AffymetrixU133a GeneChip data collected by the TRANSBIG Consortium [37,38]. These data consisted of clinical and gene expression measurements on 198 node-negative patients from five different medical centers. The data were obtained from the Bioconductor package ‘breastCancerTRANSBIG’ [39], and processed to remove duplicate probes mapping to the same Entrez Gene ID (probes with the largest variability are retained). The final gene expression data set consisted of measures on 12,701 transcripts (genes) for 198 patients. Since qRT-PCR and microarray measurements do not always correlate well, rather than validate the fitted models based on our data, we validated whether the genes selected were important for predicting breast cancer survival and recurrence. Therefore, we split the data into 1000 training (70%) and test (30%) samples, and fit Cox regression models based on genes selected for mortality and recurrence to the training sets. Separate models were also fitted based on clinical data and a randomly selected gene set of the same size, to evaluate whether our gene expression model offered improved performance relative to this information. C-index values were calculated for all models based on predictions for the test data sets. Gene expression model fitting and C-indexes calculations were also performed separately for ER+ and ER- subsets of breast cancer patients to evaluate any differences in model fit and efficacy for either ER+ or ER- carcinomas.

## Results

### Descriptive statistics and univariate survival analysis

Summary clinical and demographic information for the patient population is given in Table 1. Of the 225 cases selected, there were 28 patient records lacking some aspect of clinical information: 14 missing tumor size, 16 missing nodal status and 4 missing stage of disease. Seventy-one patients had recorded breast cancer recurrences (with 2 missing values) and 68 patients exhibited breast cancer-associated mortality. The median follow-up time was 63 months for overall survival (OS) and 57 months for disease-free survival (DFS). Seven patients that were never disease-free were omitted from Cox regressions for recurrence but not from calculations of mortality. Therefore, results from the entire study population of 225 breast carcinoma patients were utilized throughout our investigations since each case was accompanied by a breast tissue biopsy of high molecular integrity for genomic analyses.

**Table 1:** Summary statistics for clinical variables among the patient population

		**Mortality**			**Recurrence**		
**Name**	**Mean (std dev) or N (%)**	**HR (95% CI)**	**P-value**	**Adj P-value**	**HR (95% CI)**	**P-value**	**Adj P-value**
Age	59.8 (15.4)	0.99 (0.97, 1)	0.136	0.259	0.99 (0.97, 1)	0.161	0.307
Tumor size (mm)^Ϯ^	29.6 (15.1)	1.01 (1, 1.03)	0.071	0.214	1.02 (1, 1.03)	0.026	0.077
Nodes^Ϯ^							
Pos	134 (0.58)	1	-	-	1	-	-
Neg	97 (0.42)	1.78 (1.1, 2.88)	0.019	0.080	1.87 (1.17, 3.01)	0.010	0.036
Hormone therapy							
No	162 (0.7)	1	-	-	1	-	-
Yes	71 (0.3)	0.89 (0.52, 1.51)	0.657	0.986	1.05 (0.63, 1.74)	0.847	1.000
Chemotherapy							
No	152 (0.65)	1	-	-	1	-	-
Yes	81 (0.35)	2.14 (1.33, 3.45)	0.002	0.037	2.4 (1.5, 3.83)	<0.001	0.004
Radiation therapy							
No	191 (0.82)	1	-	-	1	-	-
Yes	42 (0.18)	1.56 (0.89, 2.73)	0.121	0.259	2.1 (1.25, 3.53)	0.005	0.027
Grade^Ϯ^							
1 (well differentiated)	13 (0.06)	1	-	-	1	-	-
2 (intermediate)	86 (0.42)	2.37 (0.56, 9.94)	0.239	0.419	1.54 (0.47, 5.05)	0.479	0.839
3 or 4 (poorly differentiated or undifferentiated)	105 (0.51)	2.07 (0.49, 8.68)	0.319	0.516	1.42 (0.43, 4.63)	0.565	0.913
Disease stage^Ϯ^							
1	51 (0.22)	1	-	-	1	-	-
2	143 (0.62)	1.81 (0.85, 3.86)	0.126	0.259	2.05 (0.97, 4.36)	0.061	0.161
3	27 (0.12)	3.41 (1.39, 8.35)	0.007	0.039	3.69 (1.5, 9.03)	0.004	0.027
4	8 (0.03)	4.71 (1.54, 14.4)	0.007	0.039	3.21 (0.85, 12.1)	0.085	0.198
ER/PR							
+/+	133 (0.57)	1	-	-	1	-	-
+/-	17 (0.07)	1.98 (0.82, 4.79)	0.128	0.259	3.68 (1.79, 7.58)	<0.001	0.004
-/+	30 (0.13)	2.1 (1.05, 4.21)	0.037	0.128	1.77 (0.86, 3.63)	0.118	0.249
-/-	53 (0.23)	2.26 (1.3, 3.94)	0.004	0.039	2.1 (1.19, 3.7)	0.010	0.036

Univariate hazard ratios (HRs), 95% confidence intervals (CIs) and unadjusted/adjusted p-values for disease mortality and recurrence are included. N = number, std. dev. = standard deviation. ^Ϯ^Missing values: Tumor size (6), Nodal status (2), Tumor grade (29), and Stage (4).

Hazard ratios (HRs) and 95% CIs for the association between clinical/demographic factors and breast cancer recurrence and mortality are also presented in Table 1. Tumor size, nodal status, disease stage, ER/PR status, chemotherapy and radiation therapy were significantly associated with both mortality and recurrence.

Summary information for the gene expression measurements is presented in Table 2. IL6ST exhibited the largest range in log_2_ expression measurements, from -8.23 to 12.80, while PLK1 expression had the shortest range (-5.91 to 0.48). The average interquartile range (IQR, distance between 25^th^ and 75^th^ percentiles) was 3.0, indicating that the patient’s carcinomas had a fairly broad spectrum of expression measurements (average of 3 fold difference between the 25^th^ and 75^th^ percentiles). Table 2 also provides HRs and 95% CIs for the association between the gene expression values and breast cancer recurrence/mortality. In all, expression levels of ten genes (RABEP1, PGR, NAT1, PTP4A2, SLC39A6, ESR1, EVL, TBC1D9, FUT8 and SCUBE2) gave adjusted p-values < 0.05 for association with breast cancer mortality (shaded in Table 2). Each of these genes had HRs between 0.75 and 0.90, indicating a 10% to 25% decrease in mortality risk for every 2-fold increase (doubling) in gene expression. Increased expression of these genes also correlated with a decreased risk of breast cancer recurrence, although only four (RABEP1, PGR, SLC39A6 and FUT8) exhibited significant adjusted p-values. Expression levels of only two genes (MELK, PLK1) gave positive Beta coefficients with breast cancer recurrence and mortality, with HRs between 1.20 and 1.25. In each case, the unadjusted p-values were significant, but became non-significant after adjustment for multiple comparisons.

**Table 2:** Summary statistics for gene expression levels among the patient population

		**Mortality**			**Recurrence**		
**Name**^Ϯ^	**Median (Q25, Q75)**	**HR (95% CI)**	**P-value**	**Adj P-value**	**HR (95% CI)**	**P-value**	**Adj P-value**
**RABEP1**	-0.27 (-1.43, 1.05)	0.77 (0.67, 0.87)	0.000	0.011	0.79 (0.7, 0.9)	0.000	0.042
PGR*	0.38 (-2.3, 3.09)	0.91 (0.86, 0.95)	0.000	0.014	0.92 (0.87, 0.97)	0.001	0.042
NAT1*	2.15 (-0.42, 4.89)	0.87 (0.8, 0.94)	0.001	0.020	0.88 (0.81, 0.96)	0.002	0.058
**PTP4A2**	-0.35 (-1.4, 0.95)	0.76 (0.65, 0.89)	0.001	0.020	0.79 (0.68, 0.93)	0.004	0.071
SLC39A6*	-0.38 (-2.45, 1.69)	0.87 (0.8, 0.94)	0.001	0.020	0.87 (0.81, 0.95)	0.001	0.042
ESR1*	4.27 (-0.96, 5.84)	0.9 (0.85, 0.96)	0.001	0.021	0.92 (0.87, 0.98)	0.006	0.090
**EVL**	0.68 (-0.56, 2.31)	0.8 (0.69, 0.91)	0.001	0.022	0.85 (0.75, 0.97)	0.019	0.241
TBC1D9*	0.15 (-1.87, 2.31)	0.89 (0.83, 0.96)	0.001	0.022	0.9 (0.84, 0.97)	0.004	0.071
**FUT8**	-0.51 (-1.69, 0.68)	0.82 (0.73, 0.93)	0.002	0.026	0.82 (0.72, 0.92)	0.001	0.042
**SCUBE2**	2.04 (-0.95, 4.18)	0.89 (0.83, 0.96)	0.002	0.028	0.91 (0.85, 0.98)	0.009	0.131
**GATA3**	0.87 (-1.82, 2.15)	0.89 (0.81, 0.97)	0.007	0.080	0.9 (0.83, 0.99)	0.022	0.256
**MELK**	-2.53 (-3.47, -1.54)	1.24 (1.06, 1.46)	0.008	0.088	1.19 (1.02, 1.39)	0.031	0.337
**TCEAL1**	0.45 (-0.95, 1.74)	0.83 (0.72, 0.96)	0.012	0.124	0.87 (0.76, 1)	0.057	0.502
**XBP1**	2.69 (0.73, 3.66)	0.87 (0.78, 0.98)	0.021	0.197	0.89 (0.8, 1)	0.055	0.502
**PLK1**	-2.5 (-3.32, -1.56)	1.25 (1.02, 1.52)	0.029	0.250	1.2 (0.99, 1.45)	0.058	0.502
**IL6ST**	-2.94 (-4.84, -0.45)	0.92 (0.85, 1)	0.045	0.363	0.95 (0.88, 1.02)	0.166	1.000
**DSC2**	0.31 (-0.52, 1.71)	1.1 (0.98, 1.24)	0.099	0.759	1.09 (0.97, 1.22)	0.170	1.000
**CX3CL1**	0.86 (-0.29, 2)	1.11 (0.97, 1.27)	0.123	0.890	1.07 (0.94, 1.22)	0.314	1.000
**ATAD2**	-1.18 (-1.87, -0.56)	1.08 (0.86, 1.35)	0.521	1.000	1.15 (0.92, 1.44)	0.230	1.000
**BUB1**	-3.1 (-4.23, -2.35)	1.05 (0.88, 1.24)	0.602	1.000	1.01 (0.86, 1.19)	0.869	1.000
**CENPA**	-2.18 (-3.06, -1.16)	1.07 (0.9, 1.28)	0.427	1.000	1.03 (0.86, 1.22)	0.780	1.000
**CKS2**	-1.89 (-3.32, -0.95)	0.99 (0.87, 1.13)	0.925	1.000	0.97 (0.85, 1.11)	0.678	1.000
GABRP*	3.08 (0.38, 5.36)	0.99 (0.93, 1.05)	0.698	1.000	0.97 (0.91, 1.03)	0.323	1.000
**GMPS**	-1.4 (-2.22, -0.64)	0.95 (0.79, 1.15)	0.601	1.000	0.88 (0.74, 1.06)	0.186	1.000
LRBA*	-1.71 (-3.27, 0.67)	0.99 (0.91, 1.09)	0.871	1.000	1 (0.92, 1.09)	0.986	1.000
**MAPRE2**	-1.84 (-2.84, -0.85)	1.04 (0.88, 1.23)	0.647	1.000	0.99 (0.84, 1.17)	0.921	1.000
**MCM6**	-2.27 (-3.27, -1.43)	0.96 (0.82, 1.14)	0.655	1.000	0.95 (0.81, 1.11)	0.545	1.000
**PFKP**	-2.45 (-3.42, -1.46)	1.13 (0.96, 1.33)	0.152	1.000	1.05 (0.9, 1.23)	0.539	1.000
**ST8SIA1**	-0.67 (-1.59, 0.58)	1.02 (0.89, 1.17)	0.795	1.000	1.02 (0.89, 1.17)	0.769	1.000
**TPBG**	0.68 (-0.38, 1.48)	0.89 (0.75, 1.05)	0.172	1.000	0.9 (0.76, 1.06)	0.201	1.000
**TRIM29**	-0.71 (-2.74, 1.03)	0.98 (0.9, 1.08)	0.734	1.000	0.98 (0.9, 1.07)	0.637	1.000
**YBX1**	-1.72 (-2.42, -1.12)	1.1 (0.88, 1.36)	0.413	1.000	1.04 (0.84, 1.29)	0.702	1.000

Univariate hazard ratios (HRs), 95% confidence intervals (CIs), and unadjusted/adjusted p-values for disease mortality and recurrence are included. Q25 = 25^th^ percentile, Q75 = 75^th^ percentile.

^Ϯ^Genes ordered by adjusted p-value for mortality and then by gene name. *Genes which passed the permutation threshold for significance in the multivariable model, for both mortality and recurrence.

### Multivariable cox models

**Figure 1:**
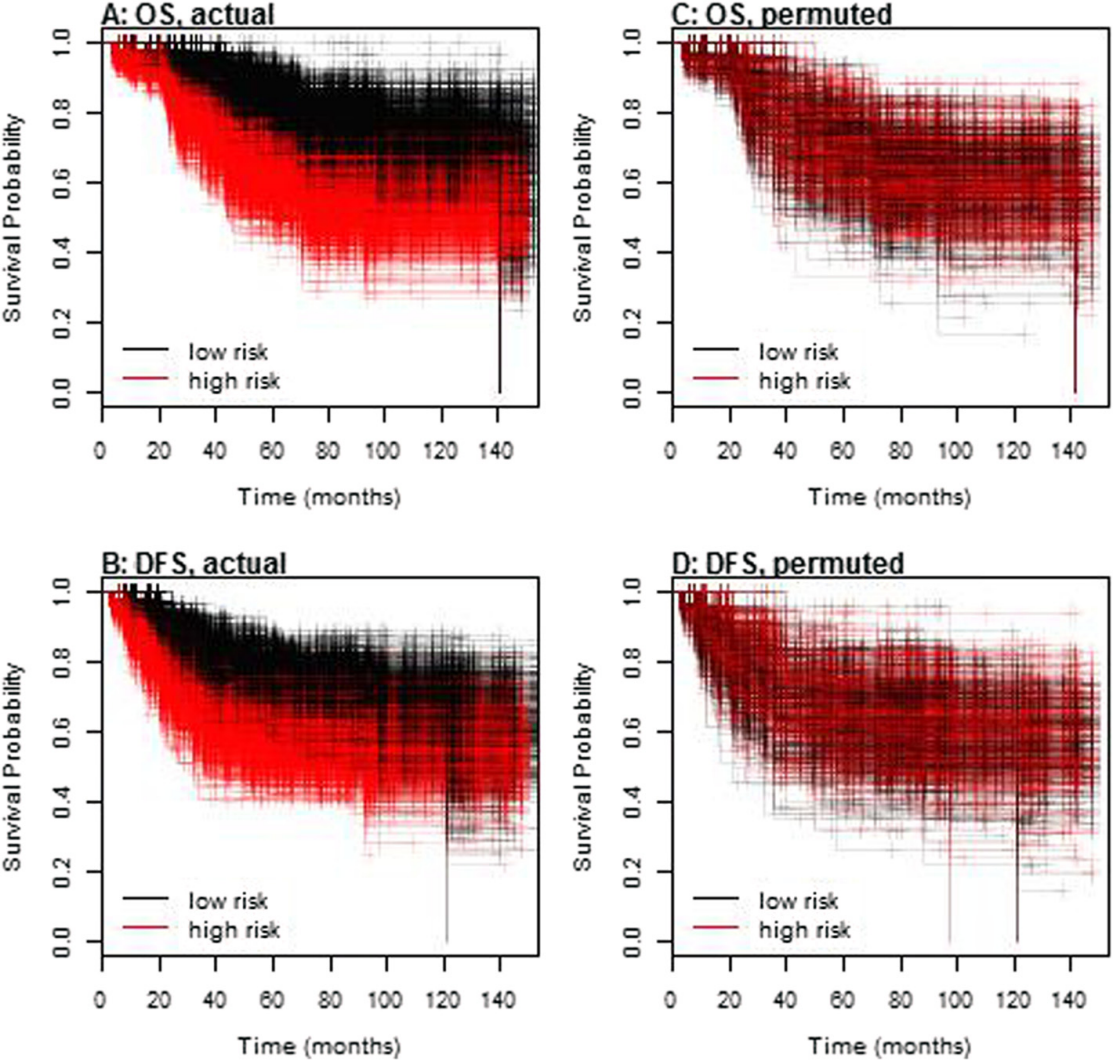
**Kaplan-Meier plots illustrating separation among the 1000 test data sets. Predictions were based on L**_**1**_**penalized (LASSO) Cox regression models fitted to each training set.** Plots **A** (OS = overall survival) and **B** (DFS = disease free survival) represent predictions of low or high risk based on actual data, while plots **C** (OS) and **D** (DFS) represent predictions based on permuted data sets.

**Figure 2:**
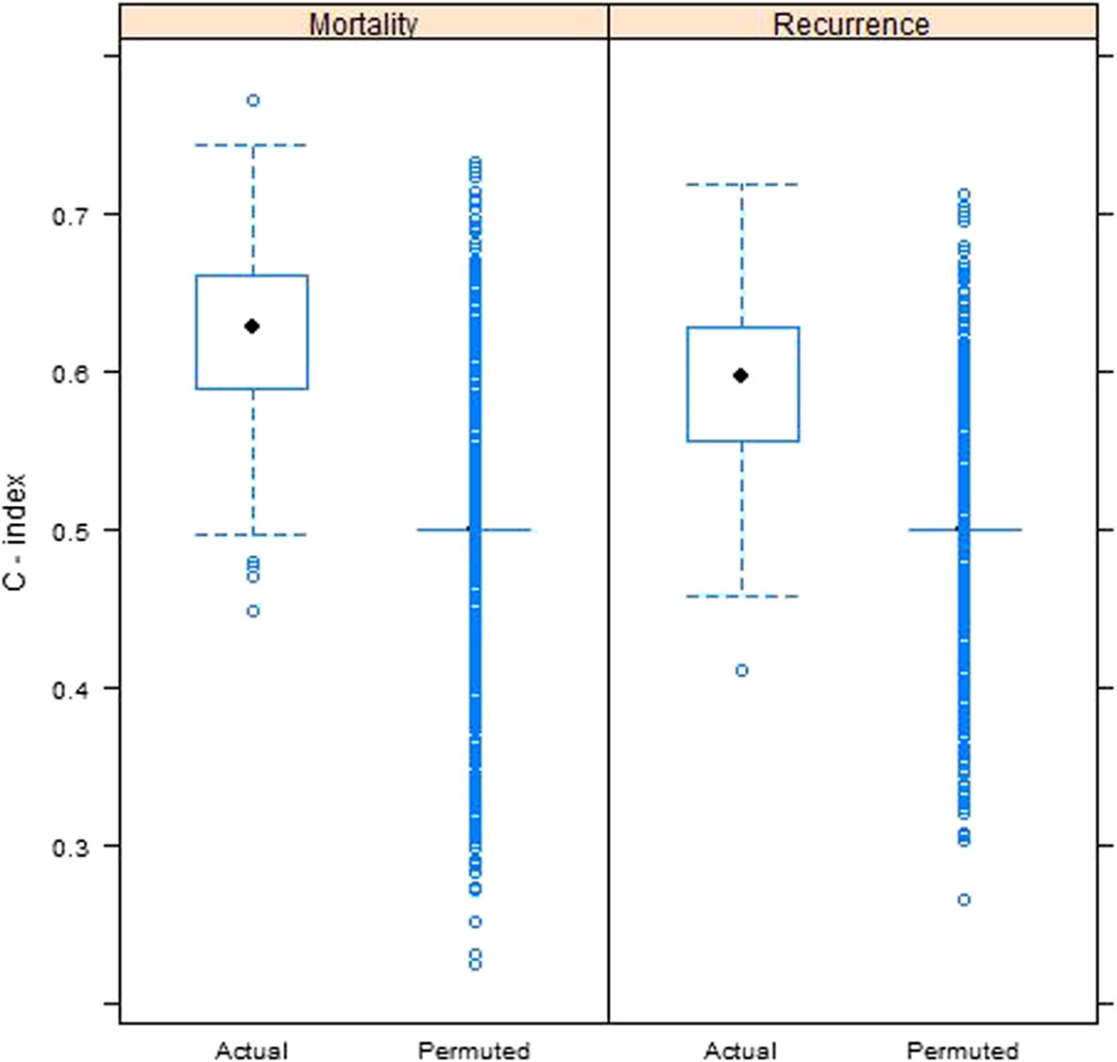
**Boxplots of C-index values of the 1000 test data sets. Predictions were made using L**_**1**_**penalized (LASSO) Cox regression models fitted to the training data sets.** Predictions made using both actual and permuted data are shown.

As indicated in the Methods section, variable selection for the multivariable model was performed by recording the percentage of times that each gene was retained in the L_1_-regulated Cox model, out of 1000 random splits into training and testing data. Based on the permutation distribution, the percent cut-off for statistical significance was 15.2% for disease mortality and 16.2% for disease recurrence. Collectively, results identified the same 7 genes selected for both disease mortality [PGR (94.4%), ESR1 (31.3%), NAT1 (30.5%), GABRP (27.7%), TBC1D9 (25.8%), SLC39A6 (20.9%) and LRBA (15.8%)] and disease recurrence [PGR (85.8%), GABRP (43.5%), SLC39A6 (38.5%), TBC1D9 (30.5%), NAT1 (30.1%), ESR1 (25.6%) and LRBA (21.1%)]. The fitted regression models on the training data sets were used to predict high and low-risk patient classes in the corresponding test sets, and Figure 1A and 1B display Kaplan-Meier plots of the high and low-risk patients for each of the 1000 test sets. Splits for predictions based on the permuted data sets are shown in Figure 1C and 1D. When considering overall survival and disease-free survival, the original data demonstrated clear separation between the two groups based on risk of breast cancer recurrence. This is in contrast to the permuted data, which demonstrated considerable overlap between high and low-risk groups. The C-index was calculated for each of the test sets, based on predictions using models fitted to the training data (see Figure 2). The median C-index values were 0.63 for disease mortality and 0.60 for disease recurrence, while the permuted distributions were centered about 0.50 as expected. Empirical 95% CIs for the C-index values were (0.50, 0.72) for mortality and (0.50, 0.68) for recurrence.

**Figure 3:**
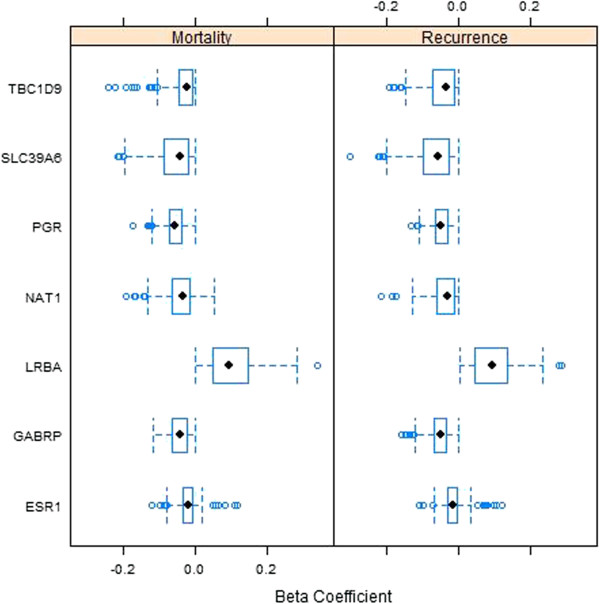
**Boxplots of Beta coefficients associated with expression of the top seven occurring genes in the Cox regression models among the 1000 training data sets, where genes were selected using the LASSO.** Left panel = disease mortality, Right panel = disease recurrence.

Figure 3 displays boxplots of the Beta coefficients from the training data models for each of the genes with frequency of occurrence in the L_1_-regulated Cox regression models above the permutation-based significance threshold (162 out of 1000 for disease recurrence, 152 out of 1000 for disease mortality). Over-expression of these genes in a breast carcinoma was predominately associated with decreased mortality (median HRs between 0.95 and 0.98). This agrees with the expression results of univariate analyses for these genes, though the magnitudes of the Beta coefficients are reduced due to the L_1_ shrinkage penalty. ESR1 expression exhibited ambiguous signs associated with its Beta coefficient for both disease mortality and recurrence, while the NAT1 levels gave ambiguous Beta coefficient signs for mortality. Both genes were omitted from the final gene expression model derived for overall survival, while only ESR1 was omitted from the disease recurrence model.

LRBA was the only gene which consistently was correlated with increased breast cancer mortality and disease recurrence when its mRNA was over-expressed in the tissue biopsy (median HR of 1.10 in both OS and DFS). Of note, the HR for LRBA by univariate analyses was 0.99 in the entire database (Table 2). The explanation for this apparent discrepancy is that LRBA is highly correlated with the other genes having higher mRNA expression associated with significantly reduced mortality (correlation between 0.31 and 0.70 for genes PGR, NAT1, SLC39A6, ESR1 and TBC1D9). The strong association of increased mortality with increased LRBA expression only resulted *after* adjustment for the presence of these other genes. This was verified by first fitting a linear regression model with LRBA as the response and PGR, NAT1, SLC39A6, ESR1 and TBC1D9 as predictors, and taking the residuals from this model. These residuals were then evaluated for association between breast cancer mortality and recurrence in a univariate Cox regression model. The resulting HR was 1.19 (95% CI 1.06 - 1.35, p = 0.005) for disease mortality and 1.23 (95% CI 1.07 - 1.41, p = 0.004) for disease recurrence. This indicates that *residual* over-expression of LRBA, which cannot be explained by the correlation between LRBA and the five genes listed above, is associated with increased risk of mortality and disease recurrence.

In a similar fashion, GABRP expression gave a non-significant univariate HR of 0.99 (Table 2), but was consistently associated with reduced mortality and disease recurrence among the LASSO selected models (median HR of 0.96 and 0.95, respectively). Conversely to LRBA, GABRP was highly negatively correlated with the same five aforementioned genes (range from -0.32 to -0.14). Cox regression models fitted using residuals from modeling GABRP expression as a response to these five genes resulted in a HR for disease mortality of 0.95 (95% CI 0.88 – 1.01, p = 0.11) and for disease recurrence of 0.93 (95% CI 0.87 – 1.00, p = 0.04). This indicates that residual over-expression of GABRP, which is unexplained by the negative correlation with these other genes, is associated with reduced risk of breast cancer mortality and recurrence.

### Comparison of gene expression models with standard clinical parameters

To determine the manner in which Cox regression models that are based on gene expression values compare with regression models based on significant clinical covariates, we again performed 1000 independent splits of the data into training and test sets. Three models were fit in each case; gene expression values only, clinical covariates only, and gene expression together with clinical covariates. The clinical covariates included stage of disease, ER status, and PR status. Tumor size and nodal status were not included since they are part of the staging system and hence redundant with disease stage. Age was not included because it was non-significantly associated with both survival outcomes. Additionally, inclusion of these parameters and tumor grade did not improve predictive accuracy on the test data sets relative to models which omitted them (data not shown).

**Figure 4:**
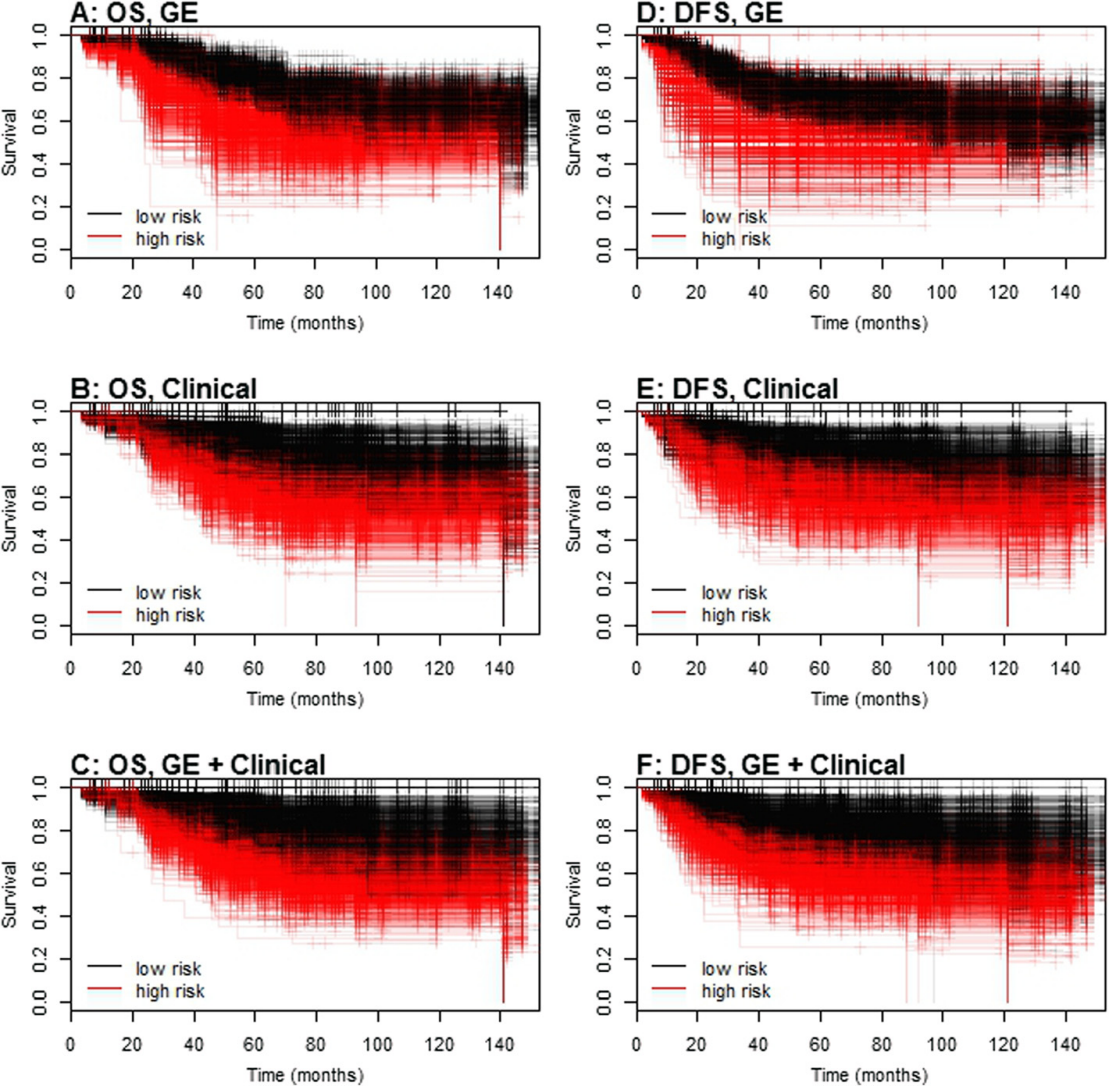
**Kaplan-Meier plots illustrating separation among the 1000 test data sets.** Predictions of low or high risk were based on Cox regression models fitted to each training set, either using gene expression (GE) only **(A** = overall survival (OS), **D** = disease free survival (DFS)**)**, clinical data only **(B** = OS, **E** = DFS**)**, or both gene expression and clinical data **(C** = OS, **F** = DFS**)**. Clinical data included for both outcomes was patient stage of disease at diagnosis (1, 2, and 3 or 4), ER status (+/-), and PR status (+/-). Genes included in both the OS and DFS models were PGR, GABRP, TBC1D9, SLC39A6 and LRBA, while NAT1 was also included in the model for DFS.

Since our goal was to compare our novel gene-signature model with the best performing clinical model, we omitted tumor grade, tumor size, nodal status, and age from the final clinical model. The fitted regression models on the training data sets were again used to predict high and low-risk patients in the corresponding test sets. Figure 4 displays Kaplan-Meier plots for the predicted low and high-risk classes of breast carcinoma patients for each set of models and outcome. Considering both overall and disease-free survival, models based on gene expression appear to better segregate the patients at high risk of recurrence, while clinical models better segregate the low-risk patients. This is corroborated by the median number of individuals identified either as low or high risk of recurrence in the test data sets, based on these models. For gene expression models, the median number of low-risk vs. high-risk patients was 55 vs. 20 and 62 vs. 12 for mortality and recurrence, respectively, while for clinical models, those numbers were 38 vs. 30 and 21 vs. 49. The smaller number of high-risk patients identified by the gene expression models in each case is reflected by the more steeply declining survival curves for these patients, relative to the clinical models. Conversely, the low-risk survival curves for the clinical models are shallower relative to the gene expression predictions. Models based on gene expression and clinical information collectively gave more balanced splits of low vs. high-risk patients, with median numbers of 31 vs. 40 for mortality and 34 vs. 37 for recurrence, respectively.

**Figure 5:**
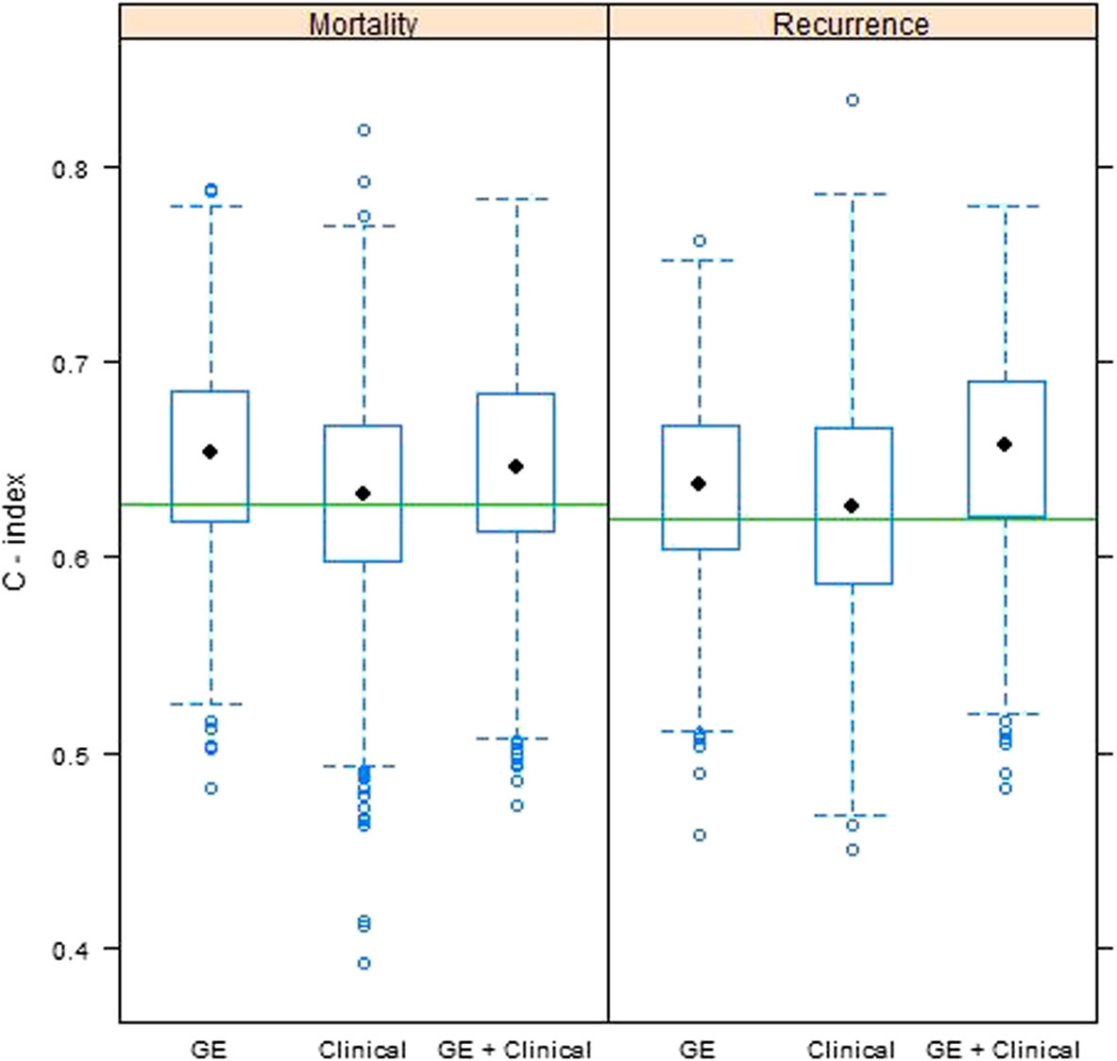
**Boxplots of C-index values for the 1000 test data sets.** Predictions were made using Cox regression models fitted to each training set. Models were derived from either gene expression (GE) data only, clinical data only, or results from both gene expression and clinical data. The green line shown on each panel represents the C-index corresponding to the 10 year Adjuvant! Online risk scores calculated for both disease mortality and disease recurrence, respectively. Clinical data included for both outcomes were patient stage of disease at diagnosis (1, 2, and 3 or 4), ER status (+/-), and PR status (+/-). Genes included in both the OS and DFS models were PGR, GABRP, TBC1D9, SLC39A6 and LRBA, while NAT1 was also included in the model for DFS.

C-index values were calculated for each test set, based on predictions from models fitted to the training data. Boxplots of C-indexes for gene expression models, clinical models, and gene expression plus clinical data models are given in Figure 5. For overall survival, the median C-index value for gene expression models was 0.65, with an empirical 95% confidence interval of 0.55 to 0.75. Comparatively, the median was 0.63 with a 95% CI of 0.52 to 0.73 for clinical models, while those for models combining clinical and gene expression results gave a median of 0.65 with 95% CI of 0.54 to 0.74. Combining gene expression and clinical information offered negligible improvement over clinical information alone, and did not improve relative to gene expression information alone. When disease-free survival was considered, the median C-index value for the gene expression models was 0.64, with 95% CI of 0.54 to 0.72. This compares to a median value of 0.63 (95% CI of 0.51 to 0.74) for the clinical models, and 0.66 (0.56 to 0.75) for combined gene expression and clinical models. The inclusion of gene expression values improved prediction relative to clinical information alone (median increase in C-indexes of 0.03), although the result was not statistically significant (95% CI for differences of -0.08 to 0.13).As a final comparison, the Adjuvant! Online (AO) ten year risks for relapse and mortality scores were calculated from known patient characteristics (i.e., age, ER status, tumor grade, tumor size, number of positive nodes) for the entire patient population. The C-index values based on predictions using these scores were 0.63 for disease mortality and 0.62 for disease recurrence (horizontal green lines in Figure 5).

**Figure 6:**
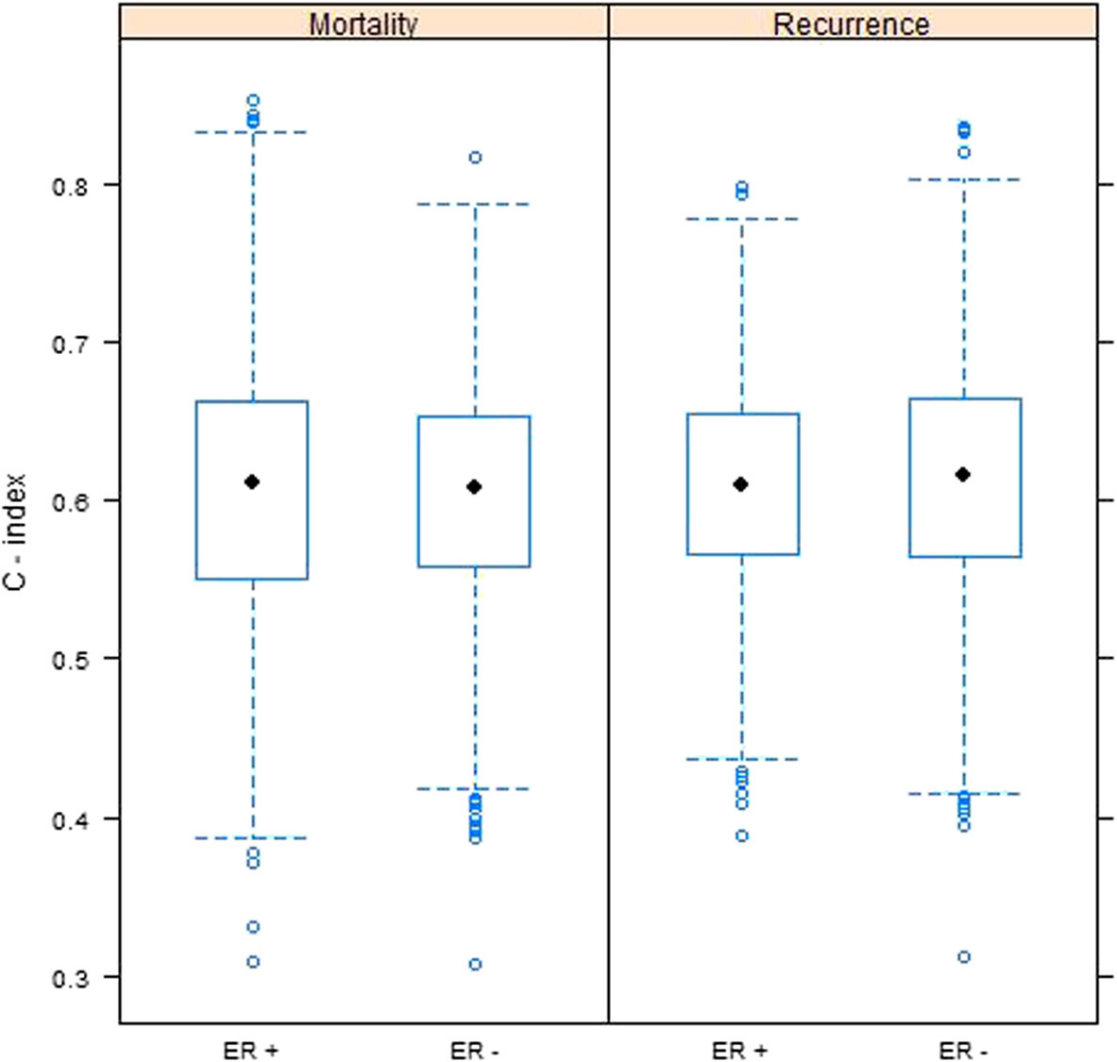
**Boxplots of C-index values for the 1000 test data sets, stratified by ER +/- status.** Predictions were made using Cox regression models fitted to each training set, derived using gene expression (GE) data. Genes included in both the OS and DFS models were PGR, GABRP, TBC1D9, SLC39A6 and LRBA, while NAT1 was also included in the model for DFS.

To evaluate whether efficacy of the gene expression signature was maintained separately within either ER+ or ER- subsets, C-index values were calculated for each test set stratified by ER+ or ER- status (Figure 6). Predictions were based on models fitted to the entire training data, rather than to models fitted separately to either ER+ or ER- subsets. This was because models fitted to all patients did not differ substantially from models fitted separately to patients with either ER+ or ER- carcinomas (data not shown). Figure 6 demonstrates that the accuracy for assessing ER+ and ER- subsets (median C-index of about 0.61 in all cases) is lower than the accuracy based on all patients (c.f. Figure 5). However, it is notable that the prognostic accuracy of the gene expression signature for ER negative patients is roughly equivalent to that for ER positive patients, for both disease mortality and recurrence. The overall lower accuracy relative to the entire cohort is attributable to the correlation between the expression of these genes and ER status of their carcinoma. Of the six genes comprising the model for DFS, only GABRP had increased expression among ER negative patients (correlation = -0.38, see Additional file 4: Figure S2). The remaining genes had positive association with ER positive status (correlation ranging between 0.34 for LRBA to 0.65 for NAT1). Due to the strong positive or negative association between the gene expression values and either ER+ or ER- status, the prognostic ability of the gene expression models is somewhat redundant with ER positive and negative subsets. This results in a decreased prognostic accuracy when evaluated separately among these subsets relative to the entire patient population.

### Validation using the TRANSBIG data

**Figure 7:**
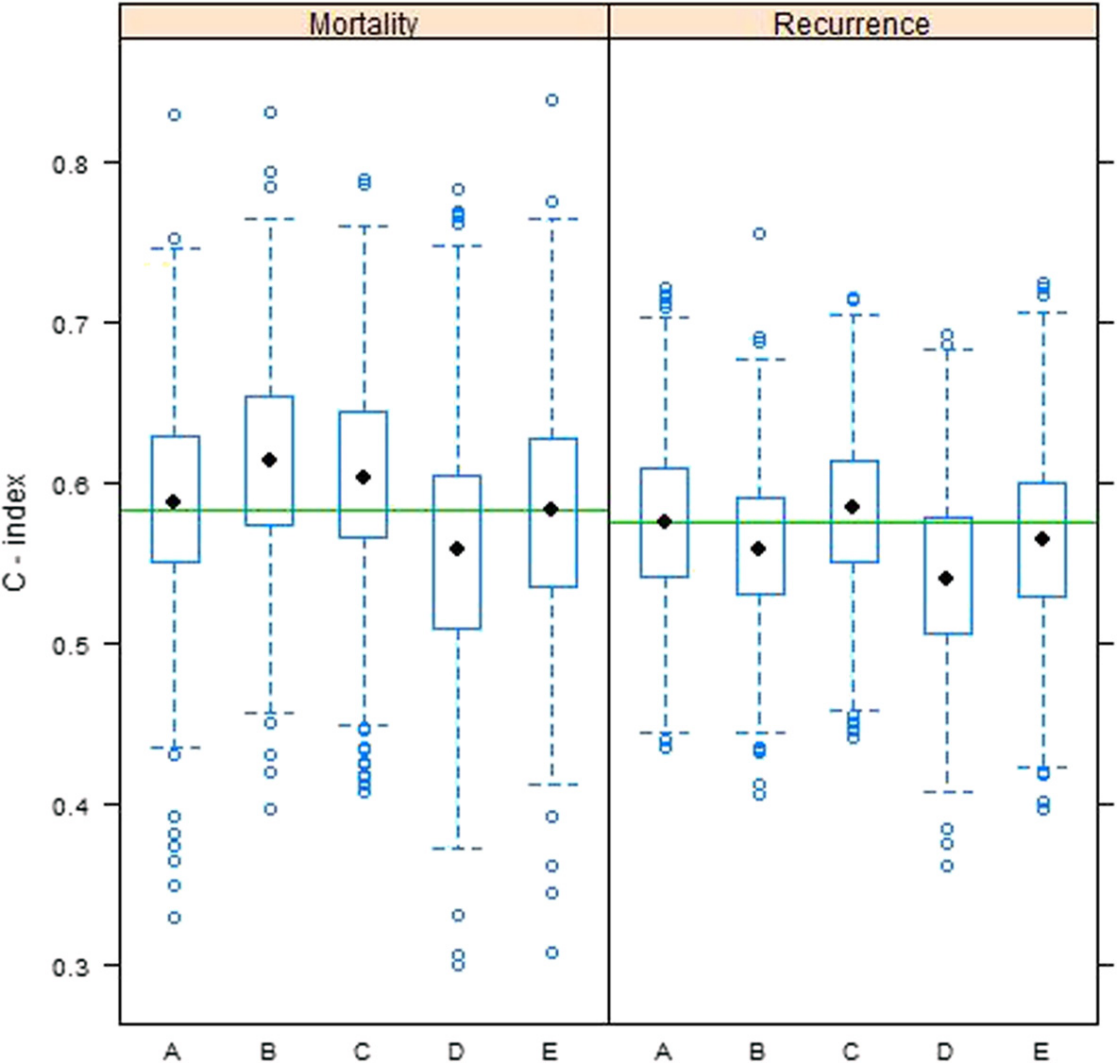
**Boxplots of C-index values for the 1000 test data sets derived from the TRANSBIG data.** Predictions were made using Cox regression models fitted to each training set. Letters correspond to the following fitted models: **A)** clinical data only (age, size and grade of tumor, ER +/- status), **B)** gene expression data (PGR, GABRP, TBC1D9, SLC39A6 and LRBA for both OS and DFS, and additionally NAT1 for DFS), **C)** clinical data plus gene expression data, **D)** randomly selected gene expression data (5 genes for OS and 6 genes for DFS), and **E)** randomly selected gene expression data plus clinical data. All five models included the medical center where the patient was seen as a covariate. The horizontal green line shown on each panel represents the C-index corresponding to the Veridex 76-gene prognostic signature [11] calculated based on the full data for both disease mortality and disease recurrence, respectively.

To validate our final selection of genes used to build models for disease mortality (PGR, GABRP, TBC1D9, SLC39A6 and LRBA) and recurrence (same gene set as for mortality, with the inclusion of NAT1), we used AffymetrixU133a GeneChip data on 198 node-negative patients collected by the TRANSBIG Consortium [37,38]. After pre-processing the data, transcripts corresponding to the genes in our models were identified and used to fit Cox regression models for disease mortality and recurrence. The following models were fit for each of the 1000 training data sets: A) clinical data only (age, size and grade of tumor, ER status), B) gene expression data (PGR, GABRP, TBC1D9, SLC39A6 and LRBA for both OS and DFS, and additionally NAT1 for DFS), C) clinical data plus gene expression data, D) randomly selected gene expression data (5 genes for OS and 6 genes for DFS), and E) randomly selected gene expression data plus clinical data. In every model, the medical center where the patient was seen was included as a covariate. C-index values for the 1000 test data sets derived from the TRANSBIG data were calculated based on predictions using Cox regression models fitted to the corresponding training set (Figure 7).

Figure 7 displays boxplots of the C-index values for the test data set predictions. The gene expression models (model B) perform quite well for disease mortality (median C-index of 0.61), but have a disappointing performance for disease recurrence (median C-index of 0.56). In both cases, the selected genes outperform a randomly selected gene set of the corresponding size (model D), though the difference is much greater for disease mortality. For disease mortality, the gene expression models also outperform the model based on clinical parameters (model A, median C-index of 0.59), though this is not the case for disease recurrence (median C-index of 0.58 for clinical model). The median C-index of our gene expression models is higher than the C-index for the Veridex 76-gene prognostic signature [11] for disease mortality (horizontal green line, C-index of 0.58), but lower than the Veridex signature for disease recurrence (C-index of 0.58). However, it should be noted that the fitted model forming the basis of the Veridex signature was calculated on an entirely independent cohort of patients, in contrast to our models which were based on training sets from the TRANSBIG data.

**Figure 8:**
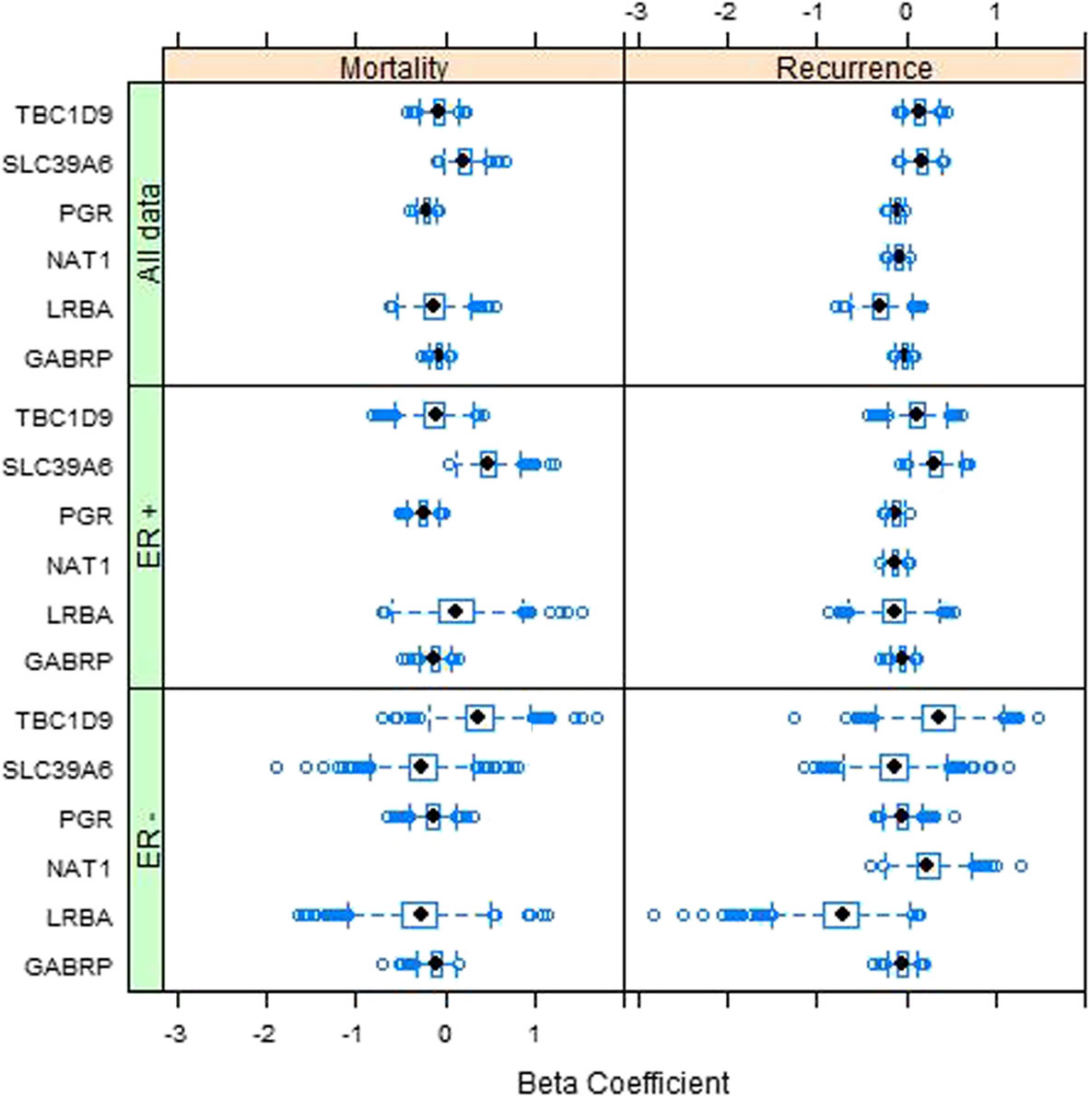
**Boxplots of Beta coefficients associated with expression of the genes in the Cox regression models fitted to the training data sets from the TRANSBIG data.** Genes included in both the OS and DFS models were PGR, GABRP, TBC1D9, SLC39A6 and LRBA, while NAT1 was also included in the model for DFS. Separate panels are given for the entire data and ER +/- subsets (rows), and for disease mortality and recurrence (columns).

Predictive accuracy of the gene expression signature was evaluated separately within either ER+ or ER- subsets of carcinomas to determine whether efficacy of the signature was maintained for these groups of patients. However, in contrast to our data, the beta coefficients for the fitted Cox models based on our selected genes differed dramatically between ER+ and ER- subsets. Figure 8 displays boxplots of the beta coefficients for the Cox regression models fitted to the training data sets from the TRANSBIG data. Coefficients for ER+ and ER- subsets of breast cancers for gene SL39A6 are in stark contrast to each other, with median estimated hazard ratios of 1.61 and 1.39 for disease mortality/recurrence for ER positive patients and corresponding hazard ratios of 0.77 and 0.87 for ER negative patients. Similarly, the median estimated hazard ratio for disease mortality associated with TBC1D9 expression is 0.89 among ER positive patients and 1.45 among ER negative patients, while the median estimated hazard ratio for disease mortality associated with LRBA expression is 1.12 in ER positive patients and 0.76 in ER negative patients. Expression of NAT1 results in a dec-reased probability of disease recurrence in ER positive patients (median hazard ratio of 0.88) but an increased probability of recurrence in ER negative patients (median hazard ratio of 1.27). Only the coefficients for GABRP and PGR are similar between ER +/- patients.

The estimated coefficients based on the TRANSBIG data for SLC39A6 and LRBA also differ considerably from the estimated coefficients based on our data (c.f. Figure 3). In particular, the estimated coefficients for LRBA based on our data are predominantly positive for both disease mortality and recurrence, whereas they are predominantly negative in both cases for the TRANSBIG data (the lone exception being disease mortality among ER positive patients). For SLC39A6 the opposite is true; coefficients based on our data are predominantly negative for both disease mortality and recurrence, whereas they are predominantly positive for the TRANSBIG data except in ER negative patients. Additional file 5: Figure S3 displays boxplots of gene expression in the TRANSBIG data for our selected genes by either ER+ or ER- status. These boxplots show good fidelity with the boxplots by ER status in our data (c.f. Additional file 4: Figure S2), which is somewhat surprising given the heterogeneity in estimated beta coefficients between the two data sets.

C-index values were calculated for each test set based on predictions in the corresponding training data set, stratified by ER status of the breast carcinomas. Additional file 6: Figure S4 displays boxplots of the C-index values for both our gene signature models and a randomly selected gene set. The signature gene expression models clearly outperform the randomly selected gene set for predicting disease mortality among ER positive patients (median C-index of 0.61 compared to 0.51), and do slightly better than the random gene set for predicting disease recurrence (median C-index of 0.56 vs. 0.52 for ER positive patients and 0.58 vs. 0.57 for ER negative patients). However, the gene signature offers no improvement relative to a randomly selected gene set for ER negative patients (median C-index of 0.55 vs. 0.56).

## Discussion

For decades, the protein products of ESR1 (estrogen receptor-α) and PGR (progesterone receptor) have been recognized as predictors of prognosis and response to hormone therapy, e.g., Tamoxifen (e.g., [20,23-25,40-42]). Therefore, it is expected that any gene subset predicting clinical behavior of breast cancer would contain ESR1 and/or PGR, and indeed this was shown earlier (e.g., [10,13,23,43]). However, several genes identified in our investigation, which were clinically relevant to breast cancer outcome, represent new targets for developing diagnostics and potentially designing targeted therapies.

The protein product of SLC39A6 (LIV-1) has been reported to transport zinc into the cytoplasm from either outside the cell or from intracellular stores [44,45]. There is increasing evidence that aberrant expression of the SLC39A family of zinc transporters promotes the epithelial-to-mesenchymal transition and leads to uncontrolled cell growth [45-47]. LIV-1 protein was shown to be regulated by estrogen, hence associated with ESR1 expression [45,48]. In addition, elevated LIV-1 protein expression in breast cancer has been associated with improved clinical outcome [48].

GABRP encodes the π-subunit of the g-aminobutyric acid (GABA) receptor, which is a transmembrane protein that is poorly understood, especially in breast tissue [49,50]. GABRP was reported to be down-regulated in 76% of breast cancers and was progressively down-regulated as tumor growth progressed, suggesting that its role may be as a tumor suppressor [50].

Wang et al. suggested that LRBA plays a role in the EGF receptor pathway [51]. In light of the fact that LRBA is a member of the WBW (WDL (WD-like)-BEACH-WD40) gene family, structural features suggest it is involved in a signaling pathway requiring interactions with other proteins, inositol phospholopids or PKA [51]. LRBA was also shown to be induced by mitogens in immune cells and over-expressed in several cancer types compared to normal tissue [51]. Of interest to our studies, LRBA was identified as co-clustering in breast tumor biopsies expressing estrogen receptor-α [52].

Although the specific intracellular functions of TBC1D9 are unknown, the TBC1 domain family of proteins is known to stimulate the GTPase activity of RAB proteins [53]. While the role of TBC1D9 is unknown in breast cancer, there is evidence that alterations in RAB GTPases play a role in progression of certain carcinomas [54].

NAT1 metabolically activates aromatic and heterocyclic amines to electrophilic intermediates that initiate carcinogenesis [55,56]. The high frequency of NAT1 acetylators genotypes are important modulators of cancer susceptibility [55]. Breast cancer tissues are reported to exhibit lower promoter methylation rates than normal breast, and DNA hypomethylation of the NAT1 gene plays a significant role in breast carcinogenesis [57]. Recently, small molecular inhibitors of NAT1 have been successful in inhibiting proliferation and invasiveness of breast cancer cells in culture [58].

Since functions of the genes in the clinically-relevant molecular signatures are involved in a variety of critical pathways in cellular differentiation and growth, no collective relationship was obvious. However, since the combined expression levels of these genes in a breast cancer biopsy appear strongly associated with a patient’s risk of recurrence and overall survival, we examined the gene set in relation to various parameters used in clinical management of the lesion. Our goal was to ascertain the competency of the gene signatures identified in our investigation to predict breast cancer outcome in comparison with that derived from conventional clinical information alone. Our objective included development of targeted gene sets (small molecular signatures) that reproducibly and objectively deduced the clinical course of breast cancer by removing the subjectivity that is often encountered from assessing multiple clinical parameters.

The variable selection strategy that we employed for determining significant genes whose expression predicted both disease mortality and recurrence involved fitting LASSO regulated Cox regression models to multiple splits of the data into training and test samples. This was accomplished as a robust approach to avoid over-fitting and produce an authentic assessment of the predictive ability of these models. To contrast, backwards elimination fitted to the entire data set retained 13 genes associated with mortality prediction and 10 genes for assessing risk of recurrence, and would undoubtedly produce an over-fitted model inapplicable with external data. Additionally, PGR, TBC1D9, and NAT1, which were found to be important predictors based on gene selection using LASSO, were not included in either backwards elimination model.

However, our approach is not without limitations. In particular, selection of genes aggregated over each gene individually, and ignored particular combinations of genes which occurred in the LASSO selected models. Assessment of these models revealed the most common model to be PGR alone (27% and 18% for mortality and recurrence, respectively), followed by various two-gene combinations which occurred at small frequencies (7% or less). Thus, there was no clear consensus of a clinically relevant gene combination based on the LASSO selected models. Another potential limitation is that all genes were retained with consistent direction of effect above the permutation testing threshold, without optimizing the number of genes to include in the final model. However, additional analyses of disease mortality by sequentially adding the most frequently occurring genes retained by LASSO resulted in little difference between models containing two to five genes (median C-indexes all 0.65). Lastly, our approach did not evaluate potential interactions between genes or non-linear effects of gene expression values. These maybe incorporated by including interaction terms in the Cox regression models, or by using a non-parametric approach [59,60]. However, the cost in potential predictive gain is increased computational burden and reduced interpretability, and the multivariable main effects Cox model is an important reference point on which to build more complicated models.

Since we used multiple testing and training splits of the *entire* data set to determine clinically relevant gene predictors, our assessment of predictive accuracy based on fitted models using these genes is slightly biased. This could be avoided by using two nested splits into testing and training samples, similar to that used in the double cross-validation method. But this approach would potentially result in a different number and combination of genes for each outer split, and would thus prevent assessment of a given combination of genes in predicting breast cancer mortality and recurrence. Another alternative would be a *single* split of the data into testing and training samples, but then the results could be sensitive to the particular split that is utilized. Our approach of using multiple testing/training splits to determine clinically relevant genes followed by a second round of multiple testing/training splits to determine predictive ability produces a model that is robust with an assessment that is only slightly upward biased. The true accuracy of our multivariable gene expression models most likely lies between the median values for the LASSO selected models and the five/six gene signature models, which is comparable to the accuracy based on clinical parameters alone.

The ability of multivariable gene expression models to accurately predict both breast cancer mortality and recurrence was evaluated relative to models based on standard clinical parameters. Further, inclusion of gene expression values was evaluated in the context of improving predictions relative to that provided by the use of clinical information alone. Results indicated that use of gene expression signatures alone was comparable to that derived from clinical information in their value for predicting both breast cancer mortality and recurrence. Thus the predictive competency of the gene signatures identified in our investigation was confirmed for assessing breast cancer outcome.

Furthermore gene expression models appeared to improve predictions for recurrence relative to those using clinical information alone (though the result was not statistically significant). Our approach for combining gene expression and clinical data was one-dimensional, in that only main effects for each variable were included in the model. However, additional investigations for disease mortality were performed to determine whether statistically significant interactions between gene expression values and disease stage, ER/PR status and treatment regimen existed. Although 11 of the 32 genes investigated exhibited significant unadjusted p-values for interaction with disease stage, none remained significant after adjustment for multiple comparisons. Nevertheless, an expanded examination of more complex models including interactions between gene expression and clinical information is warranted in future studies.

Examination of our novel gene signature separately among patients with either ER+ or ER- breast carcinomas revealed that although the predictive accuracy was diminished relative to the entire cohort, no differences in either accuracy or fitted model coefficients existed between the two subsets (Figure 6). This is in contrast to the TRANSBIG validation study, where considerable heterogeneity in both estimated model coefficients for gene expression values and predictive accuracy existed between ER+ and ER- subsets of breast cancer (Figure 8, Additional file 6: Figure S4). However, when considering the gene expression values themselves stratified by ER status, there was good agreement between our data set and that of the TRANSBIG study (Additional file 4: Figure S2 and Additional file 5: Figure S3). This suggests an alternative mechanism for the observed heterogeneity in the TRANSBIG study, perhaps involving nodal status since the TRANSBIG study consisted solely of node negative patients. However, an investigation of potential interactions between gene expression values and nodal status for predicting disease mortality and recurrence revealed no significant interactions after adjustment for multiple comparisons. Additionally, among the genes selected in our model, only NAT1 had a significant unadjusted p-value (p = 0.015) for interacting with nodal status. However, a more exhaustive model evaluation among subsets of patients stratified by clinical covariates (nodal status, ER status) is an area of future research.

Validation of selected genes using the TRANSBIG study indicated that though our gene subset performed relatively well for predicting disease mortality (median C-index of 0.61, Figure 7), the performance for disease recurrence was somewhat disappointing (median C-index of 0.56). Predictions on disease mortality did, however, compare favorably to those based on clinical data and those based on the previously published Veridex signature. It should be noted that there was considerable heterogeneity in the fitted Beta coefficients between the TRANSBIG study and our investigation, indicating that models fitted to one data set cannot be directly used to make predictions in the other data set. This can be attributed to a variety of reasons, including patient genetic and physiological heterogeneity, differences in sample processing and tissue collection, differences between qRT-PCR and microarray expression measurements, and the fact that the TRANSBIG study consisted solely of node negative patients (though, no significant interaction was found between nodal status and the genes in our novel gene signature). In light of this, the gene expression models derived in our investigation warrant further examination, validation, and possible refinement in a clinical trial setting in order to be adopted as routine clinical tests.

## Conclusions

Our goal is to identify small, clinically-relevant gene subsets to develop gene expression-based tests and gain insight into the interrelationships between these genes and clinical outcome. Based on published reports describing statistical and apparent clinical significance of various large molecular signatures of breast cancer, mRNA levels of each of 32 gene candidates were evaluated by qPCR in 225 breast carcinoma specimens. Over-expression of ten genes (RABEP1, PGR, NAT1, PTP4A2, SLC39A6, ESR1, EVL, TBC1D9, FUT8 and SCUBE2) was associated with reduced time to disease-related mortality, while four genes (RABEP1, PGR, SLC39A6 and FUT8) were associated with reduced recurrence times.

Multivariable analyses using the LASSO revealed that expression of PGR, ESR1, NAT1, GABRP, TBC1D9, SLC39A6 and LRBA of the 32 gene candidates was collectively the most important predictors for both breast cancer mortality and recurrence. Molecular signatures consisting of either five genes (PGR, GABRP, TBC1D9, SLC39A6 and LRBA) for predicting disease mortality or six genes (PGR, ESR1, GABRP, TBC1D9, SLC39A6 and LRBA) for predicting disease recurrence were identified. When taken alone, gene signatures were as effective in predicting recurrence/mortality as standard clinical parameters. However, combining the gene signature and clinical information resulted in an improvement for predicting disease recurrence relative to that derived from clinical information alone. Results from this investigation advanced the findings derived from our earlier studies [13,15,61,62], as well as those of other investigators [5-12,14] exploring breast cancer gene expression profiles. Importantly, our results identified small, biologically significant and clinically relevant gene sets in breast cancer biopsies, which predict risk of recurrence and overall survival of breast cancer patients. These molecular signatures have been sufficiently evaluated to warrant examination in a larger independent patient population (a validation cohort) such as a cooperative clinical trial, e.g., NSABP or SWOG, to verify significance for development of a routine clinical test to assess risk of breast cancer recurrence and overall survival. Prediction of the clinical outcome at the time of surgical removal of the primary lesion will facilitate improved treatment planning and disease surveillance thus enhancing individualized patient care.

## Abbreviations

qPCR: Quantitative polymerase chain reaction; RNA: Ribonucleic acid; qRT-PCR: Quantitative reverse transcription polymerase chain reaction; LASSO: Least absolute shrinkage and selection operator; RABEP1: Rabaptin, RAB GTPase binding effector protein 1; PGR: Progesterone receptor; PTP4A2: Protein tyrosine phosphatase type IVA, member 2; SLC39A6: Solute carrier family 39 (zinc transporter), member 6; ESR1: Estrogen receptor-α; EVL: Enah/Vasp-like; TBC1D9: TBC1 domain family, member 9; FUT8: Fucosyltransferase 8; SCUBE2: Signal peptide, CUB domain, EGF-like 2; NAT1: N-acetyltransferase 1; GABRP: Gamma-aminobutyric acid (GABA) A receptor, pi; LRBA: LPS-responsive vesicle trafficking, beach and anchor containing; CI: Confidence interval; LCM: Laser capture microdissection; IRB: Institutional review board; ER: Estrogen receptor; PR: Progestin receptor; CAP: College of American Pathologists; ASCO: American Society of Clinical Oncology; NCBI: National Center for Biotechnology Information; ST8SIA1: ST8 alpha-N-acetyl-neuraminide alpha-2,8-sialyltransferase 1; TRIM29: Tripartite motif containing 29; IL6ST: Interleukin 6 signal transducer; TPBG: Trophoblast glycoprotein; TCEAL1: Transcription elongation factor A (SII)-like 1; DSC2: Desmocollin 2; CENPA: Centromere protein A; MELK: Maternal embryonic leucine zipper kinase; PFKP: Phosphofructokinase, platelet; PLK1: Polo-like kinase 1; XBP1: X-box binding protein 1; MCM6: Minichromosome maintenance complex component 6; BUB1: Budding uninhibited by benzimidazoles 1 homolog; YBX1: Y box binding protein 1; GATA3: GATA binding protein 3; CX3CL1: Chemokine (C-X3-C motif) ligand 1; MAPRE2: Microtubule-associated protein, RP/EB family, member 2; GMPS: Guanine monphosphatesynthetase; CKS2: CDC28 protein kinase regulatory subunit 2; mRNA: Messenger ribonucleic acid; DTT: Dithiothreitol; dNTPs: Deoxynucleotide triphosphates; RT: Reverse transcriptase; cDNA: Complementary DNA; ACTB: β-actin; HR: Hazard ratio; FDR: False discovery rate; BH: Benjamini and Hochberg; OS: Overall survival; DFS: Disease-free survival; IQR: Interquartile range; AO: Adjuvant! Online; GABA: G-aminobutyric acid; EGF: Epidermal growth factor; WBW: WDL (WD-like)-BEACH-WD40; GTPase: Guanosinetriphosphatase; NSABP: National Surgical Adjuvant Breast and Bowel Project; SWOG: Southwest Oncology Group; TRANSBIG: A network for translational research establishedby the Breast International Group (BIG).

## Competing interests

The authors declare that they have no competing interests. The University of Louisville has filed a patent related to expression of these genes and their relationship to predicting breast cancer outcome.

## Authors’ contributions

SAA performed qPCR, interpreted data, and drafted the manuscript. GNB performed statistical analysis, interpreted data, and drafted the manuscript. JLW conceived the study, designed and interpreted experiments, and drafted the manuscript. All authors read and approved the final manuscript.

## Pre-publication history

The pre-publication history for this paper can be accessed here:

http://www.biomedcentral.com/1471-2407/13/326/prepub

## Supplementary Material

Additional file 1: Figure S1 REMARK diagram illustrating patient selection utilized in this study. Click here for file

Additional file 2: Table S1 De-identified clinical and pathological characteristics for each patient evaluated in the study. Click here for file

Additional file 3: Table S2 Gene expression results (log2 transformed) of the patient specimens evaluated in this study. Click here for file

Additional file 4: Figure S2 Boxplots of expression values for the six genes identified in the final OS (PGR, GABRP, TBC1D9, SLC39A6, and LRBA) and DFS model (as for OS with inclusion of NAT1), stratified by ER +/- status. Click here for file

Additional file 5: Figure S3 Boxplots of expression values from the TRANSBIG validation data for the six genes identified in the final OS (PGR, GABRP, TBC1D9, SLC39A6, and LRBA) and DFS model (as for OS with inclusion of NAT1), stratified by ER +/- status. Click here for file

Additional file 6: Figure S4 Boxplots of C-index values for the 1000 test data sets derived from the TRANSBIG data, stratified by ER +/- status. Predictions were made using Cox regression models fitted to each training set, separately within ER +/- subsets. Genes included in the gene signature for OS models were PGR, GABRP, TBC1D9, SLC39A6 and LRBA, while genes included in the gene signature DFS models were the same as for the OS models but additionally included NAT1. Both models were compared to models using randomly selected gene subsets of the corresponding size. Click here for file
