# Supplementary material for: Interrogating differences in expression of targeted gene sets to predict breast cancer outcome
Source: BMC Cancer. 2013 Jul 2;13:326. doi: 10.1186/1471-2407-13-326 (PMC3707751; doi:10.1186/1471-2407-13-326)
Supplement: Additional file 1: Figure S1 — REMARK diagram illustrating patient selection utilized in this study. [file 1471-2407-13-326-S1.ppt]

## Slide 1
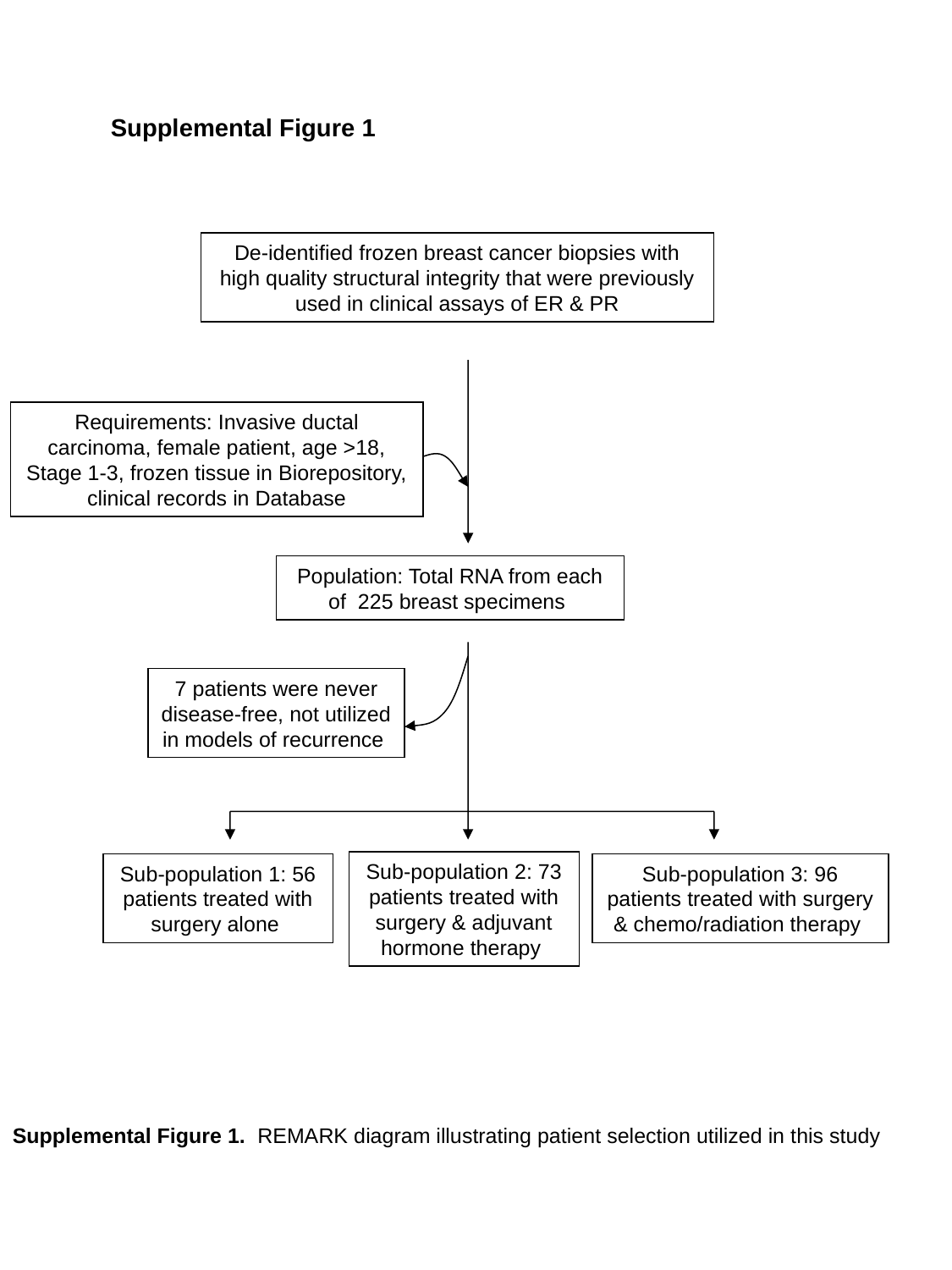

Supplemental Figure 1
De-identified frozen breast cancer biopsies with high quality structural integrity that were previously used in clinical assays of ER & PR
Requirements: Invasive ductal carcinoma, female patient, age >18, Stage 1-3, frozen tissue in Biorepository, clinical records in Database
Population: Total RNA from each of 225 breast specimens
7 patients were never disease-free, not utilized in models of recurrence
Sub-population 2: 73 patients treated with surgery & adjuvant hormone therapy
Sub-population 1: 56 patients treated with surgery alone
Sub-population 3: 96 patients treated with surgery & chemo/radiation therapy
Supplemental Figure 1. REMARK diagram illustrating patient selection utilized in this study
