# Supplementary figures and images for: Interrogating differences in expression of targeted gene sets to predict breast cancer outcome
Source: BMC Cancer. 2013 Jul 2;13:326. doi: 10.1186/1471-2407-13-326 (PMC3707751; doi:10.1186/1471-2407-13-326)

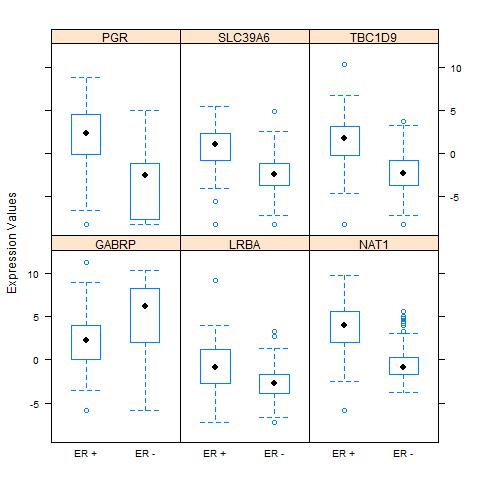

Supplement: Additional file 4: Figure S2 — Boxplots of expression values for the six genes identified in the final OS (PGR, GABRP, TBC1D9, SLC39A6, and LRBA) and DFS model (as for OS with inclusion of NAT1), stratified by ER +/- status. [file 1471-2407-13-326-S4.jpeg]

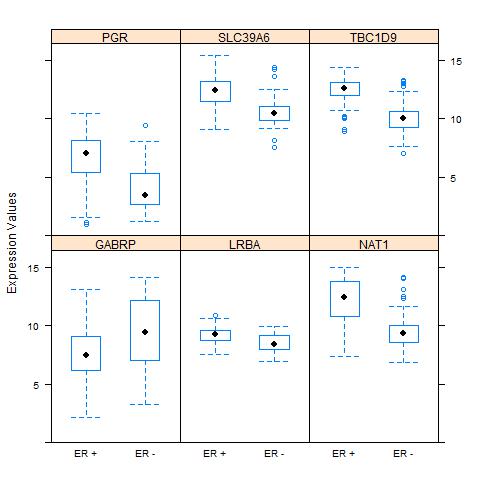

Supplement: Additional file 5: Figure S3 — Boxplots of expression values from the TRANSBIG validation data for the six genes identified in the final OS (PGR, GABRP, TBC1D9, SLC39A6, and LRBA) and DFS model (as for OS with inclusion of NAT1), stratified by ER +/- status. [file 1471-2407-13-326-S5.jpeg]

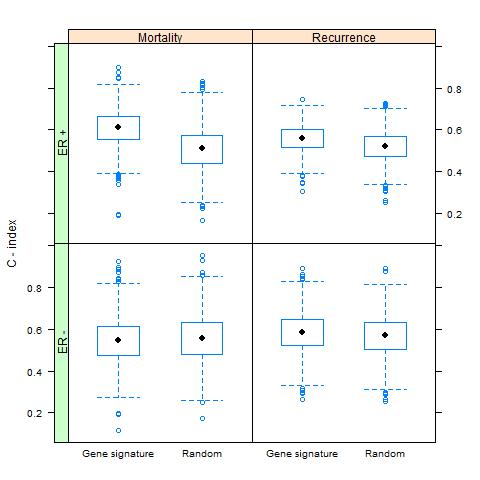

Supplement: Additional file 6: Figure S4 — Boxplots of C-index values for the 1000 test data sets derived from the TRANSBIG data, stratified by ER +/- status. Predictions were made using Cox regression models fitted to each training set, separately within ER +/- subsets. Genes included in the gene signature for OS models were PGR, GABRP, TBC1D9, SLC39A6 and LRBA, while genes included in the gene signature DFS models were the same as for the OS models but additionally included NAT1. Both models were compared to models using randomly selected gene subsets of the corresponding size. [file 1471-2407-13-326-S6.jpeg]
